# The adaptive strategies of cells in the face of CIN

**DOI:** 10.15252/embj.2023113766

**Published:** 2023-03-20

**Authors:** Zuzana Storchová

**Affiliations:** ^1^ Department of Molecular Genetics RPTU Kaiserslautern‐Landau Kaiserslautern Germany

**Keywords:** Cell Cycle, Chromatin, Transcription & Genomics, DNA Replication, Recombination & Repair

## Abstract

An increased frequency of chromosome segregation errors, known as chromosomal instability (CIN), leads to accumulation of aneuploid cells with abnormal chromosomal numbers, which impairs viability through negative effects on survival and proliferation under most conditions. Two recent papers find by independent approaches that the key to surviving high levels of CIN is reducing the instability itself, showcasing the remarkable adaptability of the chromosome segregation machinery, in particular the microtubule–kinetochore interface, and highlighting the crucial role that maintaining chromosomal stability plays in cell proliferation.

## Graphical abstract

Two recent studies show that key to surviving high levels of aneuploidy is reducing chromosomal instability via mutational adaptations of the chromosome segregation machinery.

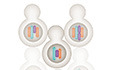

Chromosomal instability (CIN) can arise in cells with compromised chromosome segregation machinery, generating cells with variable chromosomal content. The majority of these cells may not survive, due to the detrimental effects of chromosomal gains and losses on cellular viability (Zhu *et al*, 2018). Yet, despite the negative impacts, chromosomal instability is common in cancer, where it accelerates tumor progression and acquired drug resistance (Ben‐David & Amon, 2020). Indeed, the abnormal chromosomal content, known as aneuploidy, can sometimes provide a selective advantage in stressful conditions. But how can cells survive despite the detrimental consequences associated with high CIN?

Two recent studies published in *The EMBO Journal* used budding yeasts to address these questions. Campbell and colleagues (Clarke *et al*, 2023) expanded their previously established model (Ravichandran *et al*, 2018), where chromosome segregation was compromised by reducing chromosome passenger complex (CPC) activity through deletion of Bir1, a protein required for the full CPC functionality. CPC is a key player in correcting faulty microtubule–kinetochore attachments (Carmena *et al*, 2012). In the preceding work, these authors showed that loss of Bir1 resulted in increased nonrandom aneuploidy and decreased population viability, which gradually improved with further culturing, accompanied by reduced CIN (Fig 1A and B). Here, they set up to further characterize the mechanisms underlying this adaptation. Sequencing of the surviving clones revealed further decrease of aneuploidy, but, more importantly, an accumulation of new point mutations. In 68 adapted strains, 97 nonsynonymous mutations were found. The identified hypomorphic mutations fell into four main categories: mutations in the outer kinetochore (Dam1 complex, Ndc80 complex, and Spc105, with Dam1 complex being the most mutated, Fig 1C); mutations in the CPC subunit Sli15; mutations in the mitotic checkpoint kinase Mps1 and, finally, mutations in the SCF (Skp1, cullin, F‐box) E3 ubiquitin‐ligase complex, known mainly for its involvement in the G1‐S transition.

**Figure 1:**
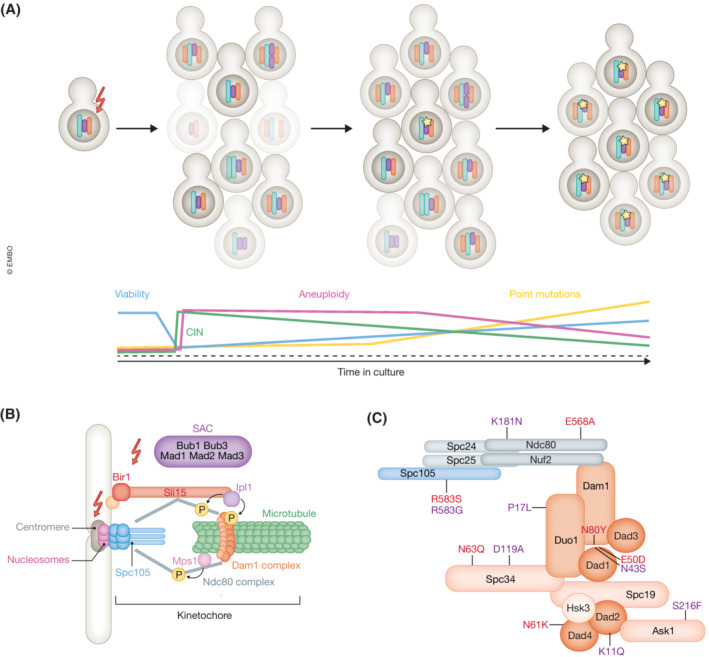
Adaptation to increased chromosomal instability occurs by adjusting the chromosome segregation machinery (A) Massive increase in chromosomal instability (CIN) results in budding yeast with aneuploidy and reduced viability. Upon prolonged cultivation, survivors with lower CIN arise. The CIN reduction occurs either via aneuploidy of specific chromosomes or via point mutations (depicted by yellow stars) affecting specific molecular machineries. Since aneuploidy compromises cell proliferation under most conditions, survivors with point mutations gradually replace the aneuploid clones. (B) Schematics of microtubule–kinetochore attachment in budding yeasts. The proteinaceous kinetochore is built on centromeric DNA occupied by specific nucleosomes. Both inner and outer kinetochore proteins (Spc105, Ndc80 complex, and Dam1 complex) help to establish the attachment to the microtubule. The connection is monitored by the chromosome passenger complex (CPC) consisting of Bir1, Sli15, and Ipl1, which ensures turnover of faulty microtubule–kinetochore attachments via phosphorylation of the Dam1 complex, and by the spindle assembly checkpoint (SAC) proteins, which delay anaphase onset. CIN was induced experimentally by (i) removing the Bir1 protein (Clarke *et al*, 2023) or by (ii) replacing the yeast histones with human homologs (Haase *et al*, 2023). (C) Most suppressor mutations were found in the proteins of the outer kinetochore (Dam1/DASH complex, Ndc80 complex, and Spc105). The individual mutations marked in red (Haase *et al*, 2023) and in purple (Clarke *et al*, 2023) demonstrate their striking convergence despite different original insults.

In the second study, Boeke and colleagues (Haase *et al*, 2023) characterized the consequences of chromosomal instability arising in cells where the yeast histone genes had been swapped for their human counterparts (Truong & Boeke, 2017). The resulting “humanized” nucleosomes impaired centromeric chromatin, as was documented by reduced nucleosome occupancy over the centromeric region and increased centromeric transcription. This defect led to high chromosomal instability, nonrandom aneuploidy, and drastically reduced viability. Also in this case, the cells eventually adapted to the conditions, and surviving aneuploid clones emerged (Fig 1A and B). During subsequent culturing, proliferation further improved, as the strains gradually returned to euploidy. Sequencing of the adapted clones revealed 60 unique nucleotide variants, which affected processes associated with the cell cycle, rRNA processing, chromatin remodeling, and chromosome segregation. Most of the mutations that suppressed the chromosome segregation errors were in genes for outer kinetochore proteins, such as the Dam1 complex, Ndc80 complex, and Spc105 (Fig 1C), remarkably overlapping with the subset of mutations identified by Clarke *et al* (2023). Although other suppressing mutations were also found, this baffling convergence of both studies on the outer kinetochore interface with microtubules indicates that the regulation of microtubule–kinetochore attachment dynamics is adjustable and offers potent routes for adaptation.

In further examinations, both groups arrived at the conclusion that the solution to diminished chromosomal stability in these clones ensued via enhancing microtubule–kinetochore turnover by weakening microtubule attachments. Through meticulous *in vivo* and *in vitro* experiments, they showed that the mutations identified by Haase *et al* (2023) disrupt the Dam1‐Dad1 interaction, thus hindering the oligomerization of the Dam1/DASH complex. Similarly, Clarke *et al* (2023) observed that the identified mutations are localized at the multimerization points of the Dam1 complex and lead to a decreased localization of the complex to the mitotic spindle *in vivo*.

Importantly, the newly arising suppressors rescued the negative effects of other mutations that compromise chromosome segregation as well, such as mutations impairing the activity of the Ipl1 kinase. In fact, some of the mutations significantly reduced the rate of chromosome segregation errors in general (Haase *et al*, 2023). This raises the question as to why these mutations did not become fixed during evolution. In other words, what is the purpose of having “less efficient” mitosis, when there are amino acid changes that could potentially lower the error rate? One explanation could be that a certain degree of reduced accuracy in chromosome segregation creates a low level of karyotypic diversity. This might be useful during evolution as a means of short‐term adaptation to stress conditions, which has been shown to occur frequently via aneuploidy (Chunduri & Storchova, 2019). However, Haase *et al* (2023) provided evidence of another potential, exciting explanation. They demonstrated that meiosis of the strains with the identified point mutations in the Dam1/DASH complex is less efficient, and this might therefore serve as the limiting factor that prevents the fixation of more “error‐free” variants of the outer kinetochore proteins.

The frequent occurrence of aneuploidy and chromosomal instability in cancer, combined with the observation that CIN negatively affects cell viability, highlights the necessity to understand how cells survive compromised genome stability. There are three potential scenarios that can be considered: cells may adapt by increasing their tolerance for aneuploidy, by optimizing their aneuploid karyotype to balance the benefits and shortcomings of aneuploidy, or by reducing the levels of CIN. While some suppressor mutations identified by the Boeke and Campbell groups have not been fully characterized yet, it seems apparent that most of the adaptations act by reducing the chromosomal instability itself. Additionally, the previously identified mutation *ubp6(E256X)*, which was shown to improve the proliferation of disomic budding yeast (Torres *et al*, 2010), did not improve the growth of chromosomally unstable yeasts lacking *BIR1*. It should be noted that disomic yeast strains contain in general less chromosomal changes than the *BIR1* lacking mutants analyzed by Clarke *et al* (2023), which readily accumulate several additional chromosomes. Thus, one can speculate that while there might be effective suppressors bypassing the aneuploid‐induced stresses upon gain of a single chromosome in otherwise stable cells, this would be insufficient to rescue the consequences of rampant chromosomal instability in the CIN strains analyzed by Clarke *et al* (2023), or by Haase *et al* (2023).

In recent years, researchers have focused on understanding the causes of chromosomal instability in cancer. Their findings so far indicate that CIN and aneuploidy in cancer may arise through diverse mechanisms and with diverse consequence (Sansregret *et al*, 2018). The works from the Campbell and Boeke groups (Clarke *et al*, 2023; Haase *et al*, 2023) highlighted here suggest that, regardless of the original causes of CIN, there may be just one universal route to adaptation to keep the levels of chromosome segregation errors in check.
